# Structure-Dependence and Mechanistic Insights into the Piezoelectric Effect in Ionic Liquids

**DOI:** 10.1021/acs.jpcb.3c07967

**Published:** 2024-02-01

**Authors:** Md. Iqbal Hossain, Haozhe Wang, Laxmi Adhikari, Gary A. Baker, Andrea Mezzetta, Lorenzo Guazzelli, Patrizia Mussini, Weiwei Xie, G. J. Blanchard

**Affiliations:** † Department of Chemistry, Michigan State University, East Lansing, Michigan 48824, United States; ‡ Department of Chemistry, University of Missouri, Columbia, Missouri 65211, United States; § Department of Pharmacy, University of Pisa, Via Bonanno 33, 56126 Pisa, Italy; ∥ Department of Chemistry, University of Milan, Via Golgi 19, 20133 Milano, Italy

## Abstract

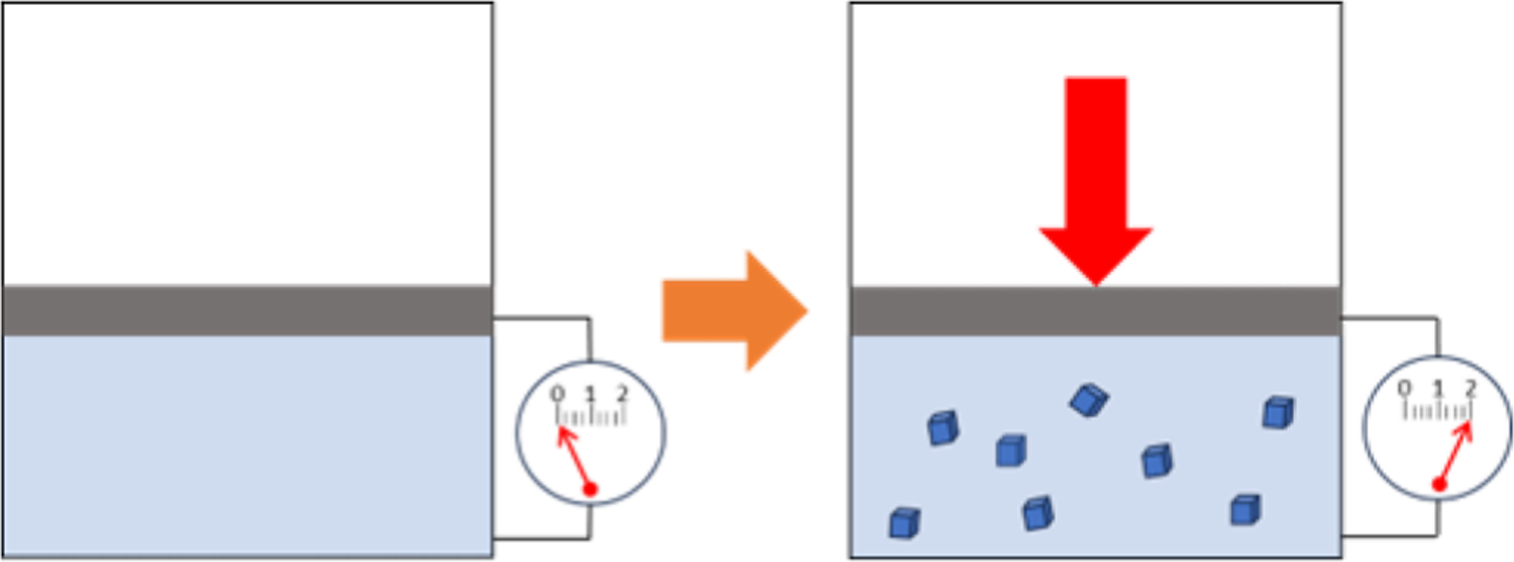

We reported recently that two imidazolium room-temperature ionic liquids (RTILs) exhibit the direct piezoelectric effect (*J. Phys. Chem. Lett.*, **2023**, 14, 2731–2735). We have subsequently investigated several other RTILs with pyrrolidinium and imidazolium cations and tetrafluoroborate and bis(trifluoromethylsulfonyl)imide anions in an effort to gain insight into the generality and mechanism of the effect. All the RTILs studied exhibit the direct piezoelectric effect, with a magnitude (*d*_33_) and threshold force that depend on the structures of both the cation and anion. The structure-dependence and existence of a threshold force for the piezoelectric effect are consistent with a pressure-induced liquid-to-crystalline solid phase transition in the RTILs, and this is consistent with experimental X-ray diffraction data.

## Introduction

The piezoelectric effect is well-known^1^ by virtue of its many applications, ranging from accelerometers and nanopositioning devices to ignition sources for appliances. This technologically important effect has been observed only in solids until recently. We have reported that two imidazolium room-temperature ionic liquids (RTILs) exhibit both the direct and converse piezoelectric effects.^2^ The discovery of the direct piezoelectric effect in RTILs was prompted by the observation of induced charge density gradients in RTILs when exposed to charged interfaces, with the gradient persisting on the order of 50 μm into the bulk medium,^3−10^ which we have since identified as the converse piezoelectric effect. Among the surprising findings in studies of the induced free charge density gradient in RTILs is that the gradient persists upon dilution of the RTILs with molecular solvents up to concentrations of ca. 30 mol % molecular solvent.^11,12^ These findings suggest that it is not molecular-scale organization but longer-range organization within the RTILs, on the order of nanometers or more, that is responsible for the effects we observe.

Organization in RTILs over length scales ranging from hundreds of nm to tens of μm has been reported by a number of groups, with the length scale and type of organization depending on the means used to detect it and the physical format of the sample.^13−22^ There is a complementary body of information on macroscopic order in the form of phase transitions in RTILs, which are dependent on temperature and/or pressure, and, as with the studies of the liquid phase RTILs, the details of the phase transitions depend on both the means by which the phase transitions are examined and the identities of the RTILs.^23−53^ The results from these bodies of work are, generally, that each RTIL exhibits system-specific behavior, with many RTILs exhibiting a liquid-to-glass phase transition with increasing pressure and some undergoing a subsequent glass-to-crystalline solid phase transition with increasing pressure. Other RTILs do not undergo a glass-to-crystalline solid phase transition with increasing pressure but do exhibit a glass-to-crystalline solid phase transition with decreasing pressure.^36^ Most of these studies have used diamond anvil cell (DAC) technology to achieve pressure control with either Raman scattering or X-ray diffraction (XRD) to characterize the morphology of the RTIL at elevated pressures.

For solid-state materials, only those that do not possess a center of inversion have the potential to exhibit a piezoelectric response. While there have been a limited number of reports where materials possessing a nominal center of inversion exhibit the piezoelectric effect, it appears that these materials distort to lift the inversion center, allowing the existence of the piezoelectric effect.^54,55^ Given that the operative model for piezoelectric solids has, to this point, provided predictions that are consistent with the experimental data for RTILs, we assume that the structural unit responsible for the piezoelectric effect in RTILs must not possess a center of inversion.^55,56^ In analogy to our dilution studies on RTILs,^3,11,12^ it is likely that the basic structural unit in RTILs is at least of nanometer dimensions, i.e., larger than individual RTIL constituent anions and cations. Any organization on the nanometer scale present in the RTILs will necessarily depend on the structures of the constituent cations and anions. In an effort to understand the relationship between the direct piezoelectric effect in RTILs and constituent ion structures, we evaluated the magnitude of the piezoelectric effect for several representative pyrrolidinium and imidazolium cations paired with tetrafluoroborate and bis(trifluoromethylsulfonyl)imide anions. Our experimental potential vs applied force data reveal an RTIL constituent-dependent piezoelectric response and a threshold force for the piezoelectric effect that likewise depends on RTIL constituent ion structures. These findings provide insight into the operative mechanism of the direct piezoelectric effect in this class of materials.

## Methods

### Materials Used

The imidazolium ionic liquids 1-butyl-3-methylimidazolium bis(trifluoromethylsulfonyl)imide (BMIM^+^TFSI^–^, >99%, <500 ppm of H_2_O, Sigma-Aldrich), 1-hexyl-3-methylimidazolium bis(trifluoromethylsulfonyl)imide (HMIM^+^TFSI^–^, 98%, Sigma-Aldrich), 1-methyl-3-octylimidazolium bis(trifluoromethylsulfonyl)imide (OMIM^+^TFSI^–^, 98%, Sigma-Aldrich), 1-butyl-3-methylimidazolium tetrafluoroborate (BMIM^+^BF_4_^–^, ≥98%, Sigma-Aldrich), and 1-methyl-3-octylimidazolium tetrafluoroborate (OMIM^+^BF_4_^–^, >97%, Sigma-Aldrich) were purified before use according to a procedure reported elsewhere.^4,57^ The pyrrolidinium ionic liquids 1-butyl-1-methylpyrrolidinium bis(trifluoromethylsulfonyl)imide (BMPyrr^+^TFSI^–^), 1-hexyl-1-methylpyrrolidinium bis(trifluoromethylsulfonyl)imide (HMPyrr^+^TFSI^–^), 1-methyl-1-methylpyrrolidinium bis(trifluoromethylsulfonyl)imide (OMPyrr^+^TFSI^–^), and 1-decyl-1-methylpyrrolidinium bis(trifluoromethylsulfonyl)imide (DMPyrr^+^TFSI^–^) were synthesized by the Baker group following methods reported earlier,^58,59^ with slight modifications.^7^ The chiral ionic liquid 1-citronellyl-3-methylimidazolium bis(trifluoromethylsulfonyl)imide (CitMIM^+^TFSI^–^) was synthesized by the Guazzelli group via a procedure reported previously^60^ and was sent to the Blanchard laboratories for these studies. After purification and prior to use, the water content of all RTILs used here was determined by Karl Fischer titration (Mettler-Toledo C10S) to be less than 50 ppm.

### Measurement of the Direct Piezoelectric Effect

The cell used to quantitate the piezoelectric effect has been described elsewhere.^2^ The cell was in the form of a cylinder-and-piston configuration, with the cylinder being made of steel and the piston made of Delrin. The piston contained a steel center electrode and had a Viton O-ring mounted near the base to create a liquid-tight seal for the RTIL contained within the cylinder. The O-ring seal was not airtight, allowing air to escape prior to the piston contacting the RTIL. The piston was 12 mm in diameter and the cylinder was 14 mm in diameter, with the O-ring providing sealed contact between the piston and cylinder. The sample size for all measurements reported here was 200 μL, resulting in a RTIL thickness of 0.64 mm.^2^ Force was applied to the cylinder, producing a potential difference between the piston electrode and the cylinder body. The force was measured by using a digital force gauge (Vetus 500N, ±1% accuracy). The apparatus was configured such that the force and potential difference are along the same axis, producing data that are related to the piezoelectric coefficient *d*_33_.

### Open Circuit Potential Measurements

Open circuit potential (OCP) measurements were made with an electrochemical bench (CH Instruments 604B). The input impedance of the bench for OCP measurements was ca. 10^12^ Ω. There was a characteristically small baseline component arising from stray capacitance that can be seen in the control measurements.^2^

### High-Pressure XRD Measurements

XRD experiments under applied pressure were carried out using a Rigaku XtalLAB Synergy-S, Dualflex, HyPix single crystal X-ray diffractometer with Mo K_α_ radiation (microfocus sealed X-ray tube, 50 kV, 1 mA, λ = 0.71073 Å). The RTIL samples were loaded inside a Diacell Bragg (S) Plus DAC manufactured by Almax-easyLab with a 600 μm culet size. The 250 μm thick steel gasket was preindented to ∼100 μm prior to sample loading. A 210 μm hole was then drilled in the center using an electronic discharge machine drilling system. Notably, no pressure-transmitting medium was employed given the liquid-state nature of the samples. The pressure was determined and monitored by the so-called “R1 line” of ruby having a fluorescence wavelength of 694.3 nm near ambient pressure and shifting to lower energies with increasing pressure, providing an accurate estimation of the pressure inside the DAC chamber. Data acquisition was executed with a 30 s exposure time per frame. To ensure reproducibility, three distinct scans were performed for each RTIL. The obtained diffraction data underwent comprehensive analysis using CrysAlisPro software. The resultant data facilitated indexing of the structure pertaining to the high-pressure phase, a process conducted utilizing GSAS-II.^61^

## Results and Discussion

As noted above, we recently reported on the direct piezoelectric effect in two imidazolium ionic liquids.^2^ In this work, we report on the magnitude of the direct piezoelectric effect in several additional RTILs, such as imidazolium cations bearing C_8_ alkyl and citronellyl functionalities and pyrrolidinium cations with C_4_, C_6_, C_8_, and C_10_ alkyl functionalities, and we compare the piezoelectric response of imidazolium RTILs with BF_4_^–^ and TFSI^–^ anions. We find that the magnitude of the direct piezoelectric effect does depend on both cation and anion structures and, significantly, that the potential vs force relationship for all RTILs is characterized by a structure-dependent threshold force. This latter point speaks to the mechanism of the direct piezoelectric response in RTILs. We consider the magnitudes of the piezoelectric response and the threshold force separately.

**Figure 1:**
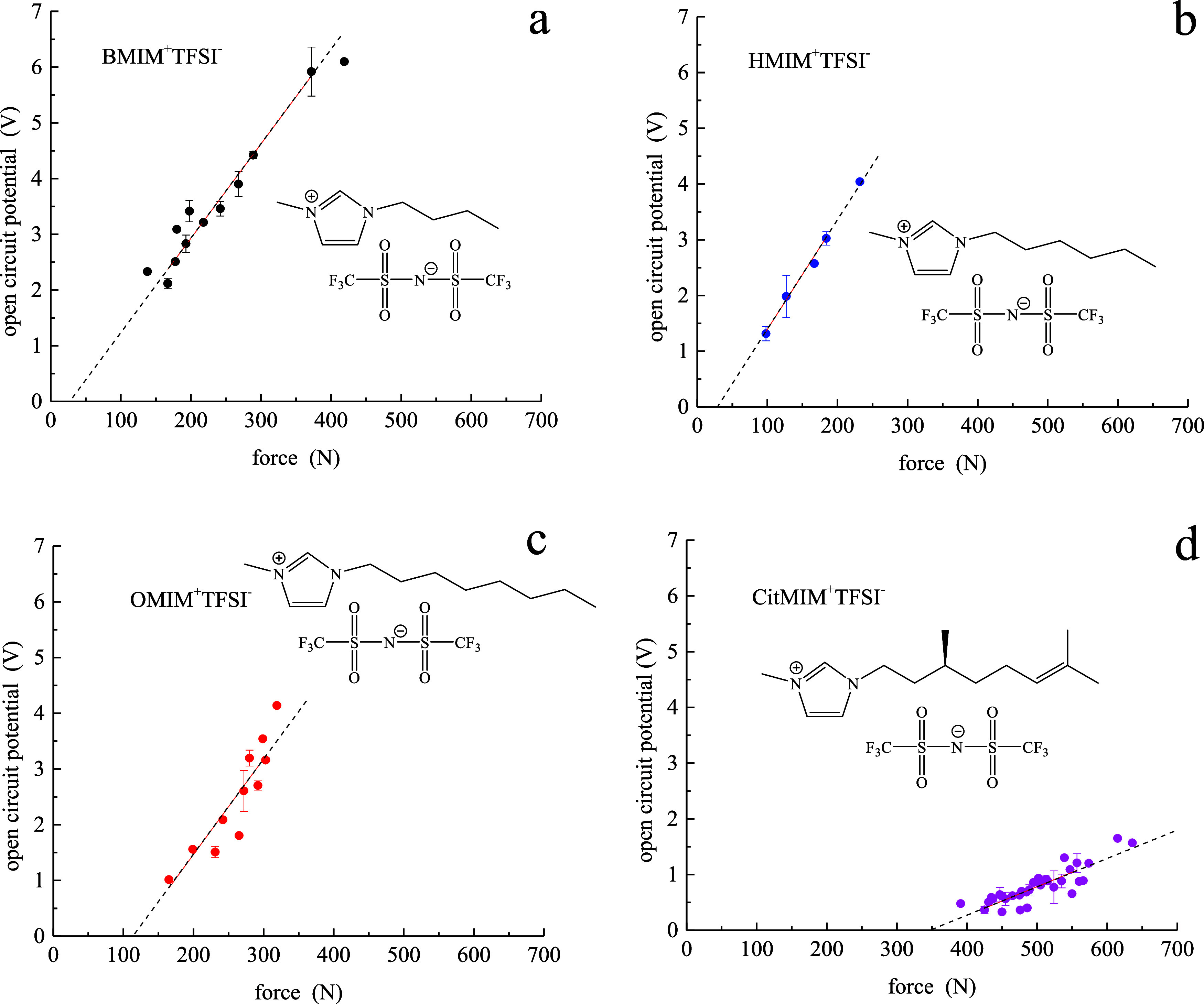
Potential (*V*) vs force (*N*) data for (a) BMIM^+^TFSI^–^, (b) HMIM^+^TFSI^–^, (c) OMIM^+^TFSI^–^, and (d) CitMIM^+^TFSI^–^. The dashed lines are least-squares fits to the data.

In the initial report, we found that the piezoelectric coefficients, *d*_33_, for BMIM^+^TFSI^–^ and HMIM^+^TFSI^–^ were the same to within the experimental uncertainty and were within a factor of 10 of *d*_33_ for quartz. To better understand the dependence of *d*_33_ on cation aliphatic chain length, we have measured the relationship between OCP and force applied for BMIM^+^TFSI^–^ (C_4_), HMIM^+^TFSI^–^ (C_6_), and OMIM^+^TFSI^–^ (C_8_) (Figure 1a–c). These data reveal that all three RTILs have the same *d*_33_ to within the experimental uncertainty (Table 1). The addition of unsaturation and a stereocenter to the imidazolium aliphatic chain, however, does exert a large effect on *d*_33_ (Figure 1d). Despite the chirality intrinsic to the CitMIM^+^ cation, the value of *d*_33_ is smaller than that for the 1-alkyl-3-methylimidazolium RTILs. We will return to a discussion of this finding, but it is clear from the data that the organization of CitMIM^+^TFSI^–^ is measurably different from that of the alkylimidazolium RTILs.

**Table 1:** Fitting Results for Potential vs Force Data for the RTILs Reported in This Work^a^

RTIL	*N*	slope (mV/N)	std. error	*Y*-int (mV)	std. error	*X*-int (N)	std. error	*d*_33_ (pC/N)	std. error
BMIM^+^TFSI^–^	12	16.9	1.7	–457	424	27	25	0.36	0.04
HMIM^+^TFSI^–^	5	19.7	1.2	–574	167	29	9	0.42	0.03
OMIM^+^TFSI^–^	11	17.2	2.5	–1964	641	115	41	0.37	0.05
CitMIM^+^TFSI^–^	31	5.2	0.8	–1807	386	349	75	0.11	0.02
BMIM^+^BF_4_^–^	18	2.3	0.3	–171	80	75	36	0.05	0.01
OMIM^+^BF_4_^–^	19	2.8	0.6	–816	325	289	131	0.06	0.01
BMPyrr^+^TFSI^–^	20	2.1	0.2	–824	79	391	47	0.05	0.01
HMPyrr^+^TFSI^–^	20	2.5	0.2	–652	76	260	35	0.05	0.01
OMPyrr^+^TFSI^–^	29	4.8	0.7	–1510	344	313	83	0.10	0.02
DMPyrr^+^TFSI^–^	24	2.7	0.6	–970	334	366	151	0.06	0.01

a *N* = number of data points; slope = potential/force in units of mV/N. Threshold force (*X*-int) in N and *d*_33_ in units of pC/N. The quantity *d*_33_ was calculated from the slope, as detailed in ref (2).

**Figure 2:**
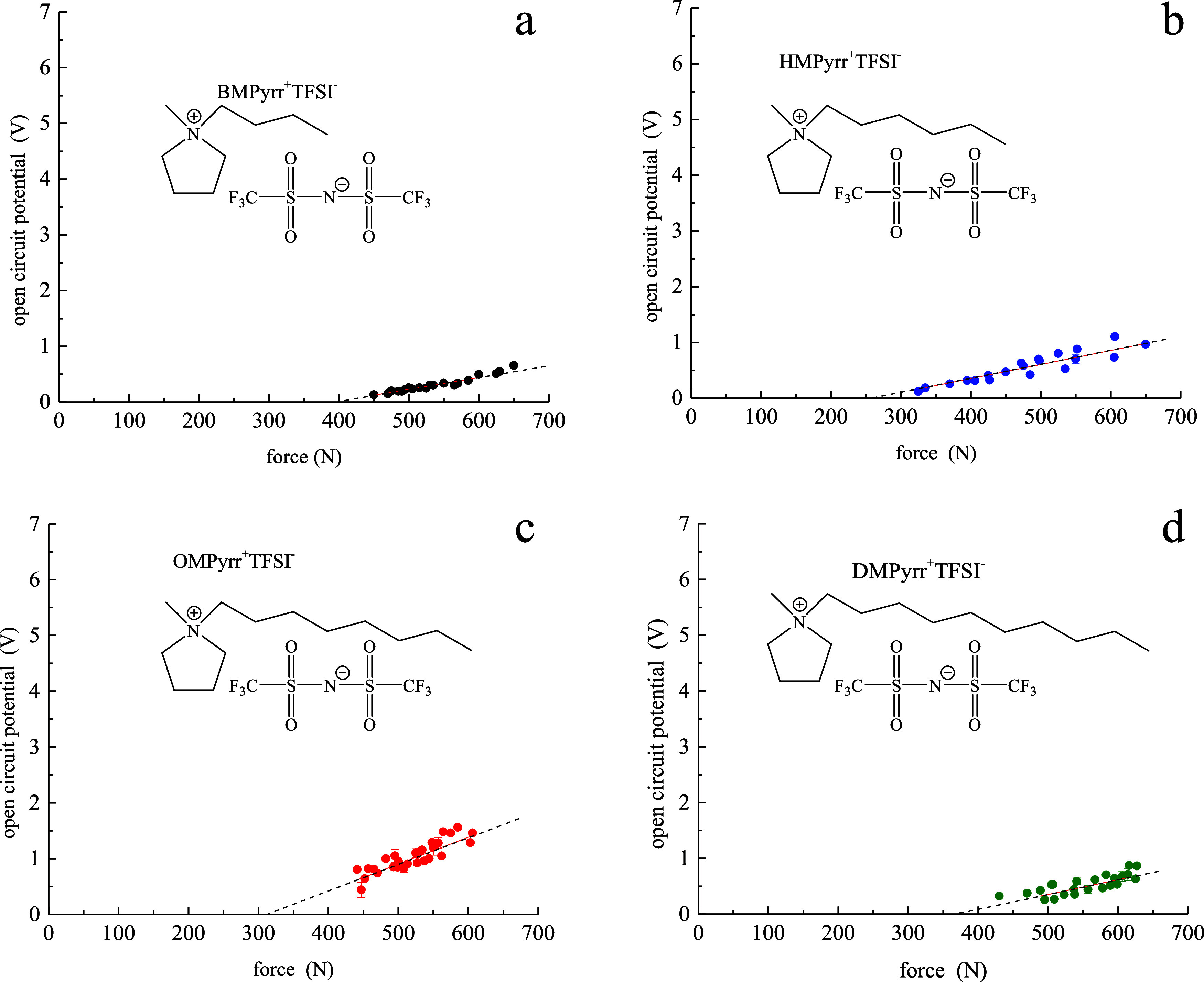
Potential (*V*) vs force (*N*) data for (a) BMPyrr^+^TFSI^–^, (b) HMPyrr^+^TFSI^–^, (c) OMPyrr^+^TFSI^–^, and (d) DMPyrr^+^TFSI^–^. The dashed lines are least-squares fits to the data.

To evaluate the role of the cation polar headgroup, we show in Figure 2 the potential vs force data for the 1-alkyl-1-methylpyrrolidinium RTILs BMPyrr^+^TFSI^–^ (C_4_), HMPyrr^+^TFSI^–^ (C_6_), OMPyrr^+^TFSI^–^ (C_8_), and DMPyrr^+^TFSI^–^ (C_10_). As was the case for the imidazolium cations, the aliphatic chain length does not appear to have much influence on *d*_33_ for the pyrrolidinium RTILs. When comparing the results for the imidazolium and pyrrolidinium RTILs, however, the imidazolium RTILs possess a *d*_33_ value ca. six times larger than that of the pyrrolidinium RTILs.

**Figure 3:**
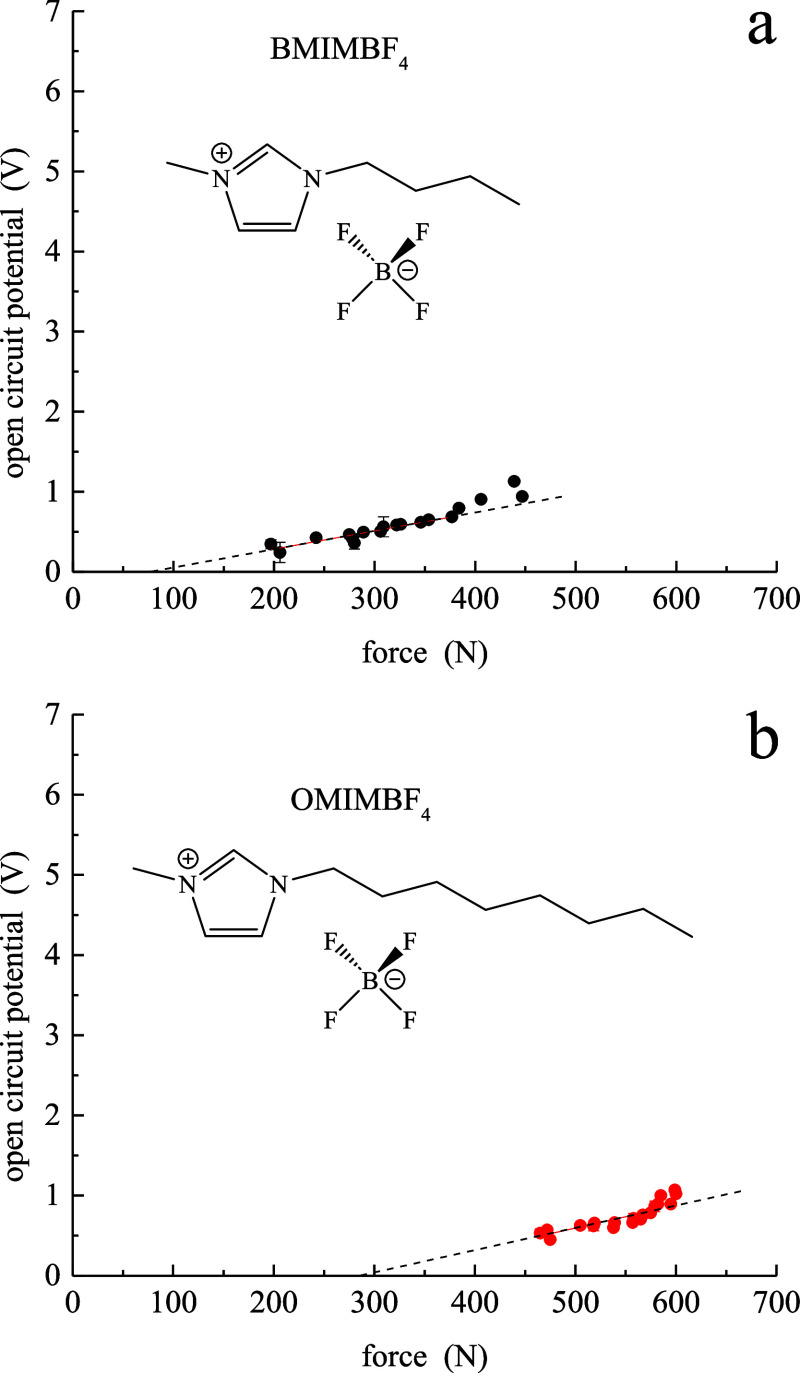
Potential (*V*) vs force (*N*) data for (a) BMIM^+^BF_4_^–^ and (b) OMIM^+^BF_4_^–^. The dashed lines are least-squares fits to the data.

The piezoelectric coefficient also depends on the RTIL anion identity (Figure 3). Comparing BMIM^+^TFSI^–^ and OMIM^+^TFSI^–^ to BMIM^+^BF_4_^–^ and OMIM^+^BF_4_^–^, the BF_4_^–^ RTILs are characterized by *d*_33_ values that are a factor of ca. 6 smaller than those of the corresponding TFSI^–^ RTILs. Again, we note the cation aliphatic chain length independence for both TFSI^–^ and BF_4_^–^ RTILs. The *d*_33_ values reported for the imidazolium BF_4_^–^ RTILs are essentially the same as those for the pyrrolidinium TFSI^–^ RTILs (Table 1). It is interesting to note that the BF_4_^–^ anion is tetrahedral, while TFSI^–^ is of lower symmetry and is known to have two stable conformers.^62−64^ It is not clear from the information we have whether one conformer is favored over another or whether the ratio of the two conformers may be pressure-dependent, but these structural issues may play a role in our observations. It is known that the two conformers are of different symmetries (C_1_ vs C_2_) and that the isomerization barrier is on the order of 30 kJ/mol.^63^

Taken together, the *d*_33_ data for the RTILs examined demonstrate several trends. The first is that the piezoelectric response is nominally independent of the cation alkyl chain length but does instead depend on the structure of the aliphatic chain. In particular, *d*_33_ for CitMIM^+^TFSI^–^, which contains a chiral cation with a branched and unsaturated tail, is significantly smaller and has a higher threshold force than that of the other 1-alkyl-3-MIM^+^TFSI^–^ RTILs. This result might be surprising because a requirement for the piezoelectric effect to be operative, at least in a solid, is that there is no center of inversion in the medium, and the use of a chiral constituent would ensure the absence of a center of inversion. However, the absence of a center of symmetry, a required condition for the piezoelectric effect, is a necessary condition for chirality but not a sufficient one. In fact, many noncentrosymmetric molecules are achiral and nevertheless exhibit piezoelectric effects. Thus, it is not straightforward that any chiral cation should necessarily result in a larger piezoelectric effect than an achiral noncentrosymmetric cation. Possibly, other factors affecting the nanoscale ordering of the RTIL ions could turn out to have a stronger impact depending on the particular structures of the cation and anion. For example, chain branching could be a determining factor and may be influential in many ways. In the case of CitMIM^+^, one of the consequences of branching is the presence of a stereocenter, making the molecule chiral. Actually, an isolated stereocenter located on an alkyl side chain, as is the case here, usually results in moderate chirality manifestations relative to other structural features, such as helical or axial stereogenic elements present within the primary molecular backbone or core.^65^ Moreover, supramolecular ordering strongly affects chirality manifestations, as made evident by circular dichroism (CD). Macromolecules made of chiral monomer units can have very large chirality manifestations when featuring a regular, tightly packed helical/foldamer secondary structure, or, on the contrary, they can exhibit negligible chirality manifestations if they possess a random coil secondary structure. For example, a polymer consisting of chiral EDOT units with short branched alkyl chains containing the stereogenic elements was reported to shift from huge chirality manifestations (sharp CD) to negligible ones (nearly zero CD) as a function of its secondary structure, modulated by experimental conditions, such as changes in the solvent proticity or increases in temperature.^66^ In any case, it cannot be assumed a priori that an ionic liquid with a cation with chirality manifestations more powerful than those of CitMIM^+^ would also necessarily display a higher piezoelectric effect. A more important factor in this respect could be the propensity of the cation or ion pair to form a highly oriented supramolecular dipolar assembly.^67^

Concerning scalar properties, branching is known to alter intermolecular interactions with respect to linear chains in many important contexts that can be exploited. For example, branching can improve oligomer solubility in polymerizations by hampering interchain interactions and chain ordering (except in cases of interdigitated assemblies of side chains),^68^ resulting in slower/thinner/smoother film formation; it can enhance polymer solubility and lower its viscosity;^69^ and it can modulate polymer strain and charge mobility as a consequence of interdigitation effects between side chains.^70^ In dye-sensitized solar cell (DSSC) sensitizers, branching can afford enhanced performance as a consequence of reduced intermolecular interactions and a diminished charge recombination rate.^71^ Liquid crystal orientation at aqueous-liquid crystal interfaces is affected by surfactant tail branching.^72^ In RTILs, branching has been reported to alter mutual solubilities with water^73^ to increase (unexpectedly) melting points and glass transition temperatures,^74,75^ increase viscosities,^73,74^ result in larger activation energies of viscous flow, lower conductivity, and decrease ionicity.^76^

It is likely that for CitMIM^+^TFSI^–^, the alteration of the intermolecular interactions and structural order caused by branching is more significant than the presence of a stereogenic element. Moreover, the above-mentioned enhancement of viscosity (i.e., resistance to deformation) might imply higher activation and a lower response for the piezoelectric effect. Increasing viscosity could result in piezoelectric vibration frequency damping.^77^ From this perspective, we might also be able to account for the very slight effect of the cation alkyl chain length, provided that they remain linear, in the 1-alkyl-3-methylimidazolium and 1-alkyl-1-methylpyrrolidinium series reported here. The effects of branching are likely more important than those of linear chain length.

A further observation is that the piezoelectric response does depend on the identity of the cationic polar headgroup or the anion. Actually, the above viscosity argument would also be consistent with the higher piezoelectric effects observed with TFSI^–^ vs BF_4_^–^ since TFSI^–^-containing RTILs generally have lower viscosity, a lower melting point, and higher room temperature conductivity than those of BF_4_^–^-containing RTILs.^78,79^ The higher piezoelectric effects observed with imidazolium vs pyrrolidinium cations for a given anion are also consistent with their viscosities. 1-Alkyl-3-methylimidazolium RTILs with TFSI^–^ are systematically less viscous than their 1-alkyl-1-methylpyrrolidinium counterparts. BMPyrr^+^TFSI^–^ has also been characterized as having less charge localization and organization than those of BMIM^+^TFSI^–^, which was characterized by a greater extent of directional order.^80^ If this finding is true, it could imply a much more efficient dipole arrangement for piezoelectric effects in the imidazolium RTILs than that in the pyrrolidinium RTILs. A lower order in the pyrrolidinium RTILs would also be consistent with the many degrees of conformational freedom of pyrrolidinium aliphatic cations, resulting in many conformers and in systematically higher pressure for glass transition than that for the more rigid, planar aromatic imidazolium cations, as has been reported in a comparative study.^81^ In that study, the liquid “structure” of the pyrrolidinium RTIL under external pressure tends to resist shear stress through cooperative conformational changes of the cation and the anion.

With these results in mind, we turn to a discussion of another important feature of the data presented in Figures 1–3. Specifically, regression of the potential vs force data for each RTIL reveals that there is a threshold force required for the piezoelectric response to manifest. This feature was not apparent in our initial report based on the relatively small threshold force value for the 1-alkyl-3-methylimidazolium^+^TFSI^–^ RTILs, but the acquisition of more data and the use of regression analysis show clearly the existence of a threshold force. With a greater range of RTIL piezoelectric studies now in hand, the threshold force is clearly seen to depend on the RTIL cation and anion structures (Table 1). The presence of this feature in the data is important because it provides a means of reconciling the piezoelectric effect in RTILs with the piezoelectric effect in solid-state materials.

**Figure 4:**
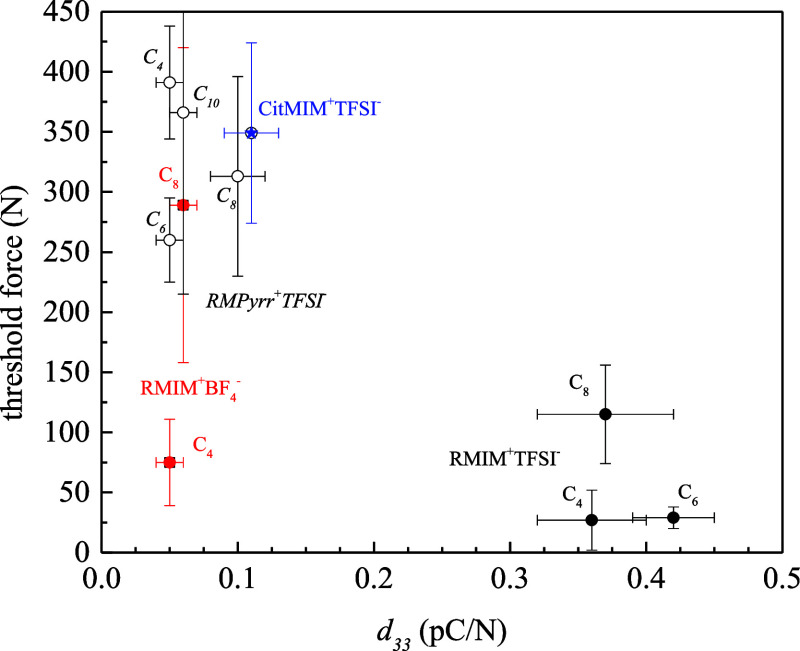
Threshold forces for the piezoelectric response as a function of *d*_33_. Filled black circles: alkylMIM^+^TFSI^–^, red squares: alkylMIM^+^BF_4_^–^, open circles: alkylPyrr^+^TFSI^–^, and blue star: CitMIM^+^TFSI^–^. Cation aliphatic chain lengths are as indicated.

It is further interesting to note that the magnitude of the piezoelectric coefficient, *d*_33_, and the threshold force for the piezoelectric effect appear to be correlated, with a high *d*_33_ corresponding to the lowest threshold force, and vice versa. Plotting threshold force versus *d*_33_ (Table 1) yields two clusters (Figure 4). BMIM^+^BF_4_^–^ is the only apparent anomaly, possibly attributable to the reported cation tail-curling effect of alkylmethylimidazolium RTILs under applied external pressure in the presence of BF_4_^–^ counteranions, which is not seen with TFSI^–^.^16^ One cluster contains the RTILs characterized by larger *d*_33_ and requires a relatively low threshold force, while the other cluster is characterized by smaller *d*_33_ and entails a higher threshold force to activate the piezoelectric response.

There is a substantial body of literature on pressure-induced phase transitions within RTILs, with the majority of the data being based on XRD and/or Raman scattering.^23−25,27−30,33−44,46−53,82^ In that literature, the most commonly observed phase transition is from liquid to glass with increasing pressure, although there is evidence for crystalline phases under certain conditions, with the formation of such phases sometimes occurring during depressurization and being kinetically mediated.^36,41,49^ A significant limitation of that literature, however, is that the range of pressures studied is typically much higher than we apply to the RTILs to observe a piezoelectric response. Given the experimental geometry of our piezoelectric measurement system, typical pressure ranges we can access are on the order of 0.001 to 0.050 GPa, whereas the pressure ranges accessed by DACs are typically in the range of 0.4 to 3 GPa. Given the variety of conditions, RTIL structures, and details of pressure-induced phase transitions reported for RTILs in the literature, it is important for us to determine whether a liquid-to-crystalline solid-phase transition is observed for the RTILs studied here.

**Figure 5:**
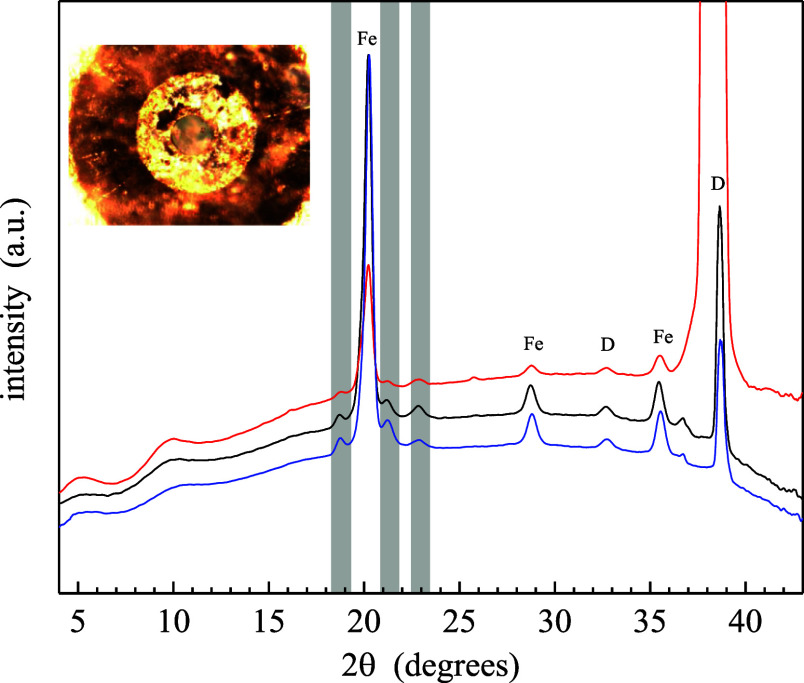
XRD pattern of HMIM^+^TFSI^–^ (blue), HMPyrr^+^TFSI^–^ (red), and CitMIM^+^TFSI^–^ (black) in a DAC. Peak locations for diamond (D) and iron (Fe) are indicated. The three peaks in the shaded windows are associated with the RTILs. Inset: photograph of a DAC loaded with RTIL.

Toward this end, we have examined HMIM^+^TFSI^–^, HMPyrr^+^TFSI^–^, and CitMIM^+^TFSI^–^ using XRD (Figure 5). There are a number of peaks in these data, including those associated with the RTILs, diamond, ruby, and steel gaskets. The fact that there are discrete peaks associated with the RTILs demonstrates that the RTILs have undergone a liquid-to-crystalline solid pressure-induced phase transition. We assert that a crystalline solid-phase RTIL is the structural entity responsible for the direct piezoelectric effect. This finding is consistent with the accepted model for the piezoelectric effect, and the XRD data provide some insight into the possible structures of the RTIL crystals. We consider these data in detail below.

The XRD data showing discrete peaks that are assigned to the RTILs can be indexed to obtain the lattice parameters. These parameters are summarized in Table 2, and they provide some important structural insight. We present in Table 2 the best two indexing results for the RTIL unit cells. Based on fitting parameters alone, it is not possible to distinguish between the two possibilities. However, when these fitted results are examined in the context of other data, it is possible to gain some additional insight. We show in Table 3 the hydrodynamic volumes and the maximum RTIL cation length for the systems examined by XRD. The hydrodynamic volumes were calculated according to the method of van der Waals increments,^83^ and the maximum lengths of the RTIL cations were calculated using molecular mechanics. We recognize that the hydrodynamic volume calculations may not be in exact agreement with other methods of estimating molecular volume, but this method has proven to be useful and accurate when applied to solution-phase molecular motion measurements. The maximum lengths of the RTIL cations are calculated by assuming all-trans aliphatic chains. These data, when viewed in the context of the fitted unit cell volumes, allow the elimination of some possible fits and the estimation of the number of molecules in the unit cell. For HMIM^+^TFSI^–^, either fit could be valid based on the HMIM^+^ length. For unit cell 1, the volume/molecular volume was 2.06 (∼2), and for unit cell 2, the ratio was 2.47 (∼2.5). For reasons of symmetry, unit cell 1 would correspond to two HMIM^+^TFSI^–^ ion pairs, and unit cell 2 would correspond to five HMIM^+^TFSI^–^ ion pairs. For HMPyrr^+^TFSI^–^, for fitted unit cell 1, the unit cell-to-molecular volume ratio was 2.58 (∼2.5), and for unit cell 2, the ratio was 1.29 (∼1.33). Based on cation length, either fit is plausible. Unit cell 1 would correspond to five RTIL ion pairs and unit cell 2 would correspond to four ion pairs in the unit cell. For CitMIM^+^TFSI^–^, the cation maximum length is not consistent with fitted unit cell 2, but it is consistent with unit cell 1. For this unit cell, the unit cell-to-molecular volume was 2.35 (∼2.33), corresponding to seven RTIL ion pairs within the unit cell. While these data collectively place limits on the structure and unit cell size of the RTIL crystalline solid phases, another constraint that must apply for a piezoelectric material is that the crystalline material cannot possess a center of inversion. While not a requirement, it is more likely that unit cells containing an odd number of ion pairs do not possess a center of inversion.

**Table 2:** XRD Lattice Fitting Results

RTIL	fitted unit cell	*a* (Å)	*b* (Å)	*c* (Å)	α (deg)	β (deg)	γ (deg)	*V* (Å^3^)
HMIM^+^TFSI^–^	1	10.459	10.459	6.700	90	90	90	732.95
	2	10.152	10.152	9.825	90	90	120	876.84
HMPyrr^+^TFSI^–^	1	9.641	8.198	12.127	90	90	90	958.45
	2	11.387	6.032	8.263	90	122.15	90	480.51
CitMIM^+^TFSI^–^	1	8.047	9.288	13.102	90	90	90	979.30
	2	8.291	9.291	7.403	90	90	120	440.73

**Table 3:** Calculated RTIL Hydrodynamic Volumes and Maximum RTIL Cation Lengths

RTIL	hydrodynamic volume (Å^3^)	maximum cation length (Å)
HMIM^+^TFSI^–^	355	12.34
HMPyrr^+^TFSI^–^	372	10.66
CitMIM^+^TFSI^–^	416	12.48

It is important to view these results in the appropriate context. As noted above, the pressure range we access to observe the direct piezoelectric effect is substantially lower than that we access using the DAC to acquire XRD data under pressure. As such, the evidence for RTIL crystal formation is important, but the data in Figure 5 represent a limiting structural case in terms of the piezoelectric activity we observe. It is also useful to keep in mind that a high degree of crystallinity is not required for the piezoelectric effect. Certain soft materials are known to exhibit a (weak) piezoelectric response.^84^

The literature on pressure-dependent phase transitions in RTILs has shown that, for BMIM^+^PF_6_^–^, there is a pressure-dependent progression in the crystalline forms of the RTIL,^31^ and such behavior is not specific to this RTIL. Thus, the crystalline forms we report from our XRD data are not necessarily the same as the crystalline forms responsible for the direct piezoelectric response. Despite the absence of a direct structural connection, the functional form of the potential vs force data (Figures 1–3) can offer some additional information. The data suggest by virtue of their linearity that the relevant phase transition is a first-order phase transition. The acquisition of data over a greater range of applied pressure will be helpful in resolving more details about the nature of the relevant phase transition and whether multiple, pressure-dependent crystalline phases can be accessed.

At present, several issues remain under investigation. Among them is resolving the size(s) of the crystalline structures formed in the RTILs and the properties of the macroscopic system upon the application of comparatively low pressure. It remains to be resolved whether the RTIL, upon application of pressure, forms solid crystalline domains in a liquid matrix or a glass matrix. Experimentally, the observation of the direct piezoelectric effect is consistent with the crystalline domains responsible for the effect being oriented anisotropically with respect to the axis of the applied force. Any rerandomization of crystalline domains that would occur over time as a consequence of their existence in a liquid matrix would cause a loss of induced potential under constant pressure, and this is not seen. The pressure-induced potential appears to remain at a relatively constant level during the application of nominally constant pressure. Interestingly, the viscosity-dependence of the *d*_33_ data (vide infra) would seem to suggest the importance of mobility in the crystalline domain formation process.

The physical domain sizes of the piezoactive crystalline structures must also be related in some manner to the organization in the RTIL associated with the converse piezoelectric effect. The smallest structural unit required for the piezoelectric material to be operative is larger than either individual ions or ion-paired units. We know that the converse piezoelectric effect in RTILs is associated with a charge displacement gradient, ∇·D, that persists for ca. 50 μm.^2,4,5,8,10^ The organization that is important to the direct piezoelectric effect is thus greater than the molecular scale, with an upper bound determined by the length scale of ∇·D. In fact, the functional relationship between the magnitude of the direct and converse piezoelectric effect(s) in RTILs remains to be quantified. Structural organization on the μm length scale is unprecedented in a “normal” liquid phase medium and would be readily detectable through scattering studies, and there is currently no experimental evidence in support of such long-range structural order in a bulk RTIL.

Another issue that requires resolution is the nature of the solid phase under the conditions of increasing and decreasing pressure. It is known that for some RTILs, the application of increasing force leads to a liquid-to-glass phase transition, which undergoes relaxation through crystalline phases with decreasing force.^36,41^ The experimental data reported here in Figures 1–3 appear not to be consistent with this case. The piezoelectric potential is observed upon the application of increasing force rather than as a result of reducing force. Given the requirement of noncentrosymmetric media for the existence of the piezoelectric effect and the direct relationship between piezoelectric potential and applied (increasing) force, the phase transition we observe must involve the pressure-induced formation of crystalline domains. Further work is required, with an emphasis on direct examination of the crystalline structure(s) of the domains formed under comparatively low pressure conditions, and this is presently underway.

The experimental data (Figures 1–3) demonstrate that *d*_33_ depends on the structures of the RTIL cations and anions. Because the domain sizes of the piezoactive structures remain to be resolved, the interplay between molecular-scale RTIL cation and anion structures and the formation of crystalline domains cannot be addressed at this point. Thoughtful design of RTIL cations and anions and characterization of their piezoelectric response will provide more insight into the structural dependence of this interesting effect.

## Conclusions

The data we report here have demonstrated several important points. First, the identification of pressure-induced liquid-to-crystalline solid phase transitions in RTILs allows reconciliation of the direct piezoelectric effect in these materials with the established model of the piezoelectric effect in solids, at least for the direct piezoelectric effect. Second, the piezoelectric effect in ionic liquids is a relatively general effect for this class of materials and does not appear to be restricted to a small subset of structures. In fact, some RTILs may exhibit the coexistence of a liquid phase and a crystalline solid phase, depending on the RTIL constituents.^85^ This finding is important because it implies the utility of this class of materials for technological applications involving the piezoelectric effect.

Our findings also demonstrate that the magnitude of the piezoelectric response of RTILs does indeed depend on the identities of the cation and anion constituents, thereby providing useful hints toward rationalization and optimization of the effect. Larger values of *d*_33_ correlate with lower activation thresholds with the TFSI^–^ anion, and alkylmethylimidazolium cations exhibit larger *d*_33_ values than those of the less rigid alkylmethylpyrrolidinium cations. For both cations, the *n*-alkyl chain length does not appear to affect *d*_33_ very much. The comparatively modest *d*_33_ of CitMIM^+^TFSI^–^ relative to alkylMIM^+^TFSI^–^ RTILs shows that chirality, albeit implying noncentrosymmetry (i.e., a necessary condition for the piezoelectric effect), does not necessarily manifest a larger piezoelectric response. In this case, the effect of branching on scalar properties appears to be much more relevant than the incorporation of a stereogenic element.

In addition to being of fundamental interest, the observation of the direct and converse piezoelectric effects in RTILs is likely to be of practical importance in areas where traditional solid-state piezoelectrics are limited. For example, piezo-pneumatic devices and highly damage-resistant liquid-phase linear and nonlinear optics could be realized based on the converse piezoelectric effect, and large-format, spatially addressable charge-generation devices based on the direct piezoelectric effect would benefit from the fluid properties of RTILs.
